# Treatment of Graves' Disease Associated With Severe Neutropenia

**DOI:** 10.7759/cureus.21014

**Published:** 2022-01-07

**Authors:** Soukaina Laidi, Imane Motaib, Saloua Elamari, Said Anajar, Asma Chadli

**Affiliations:** 1 Endocrinology, Diabetology, Metabolic Diseases and Nutrition, Mohammed VI University of Health and Medical Sciences (UM6SS), Casablanca, MAR; 2 Otorhinolaryngology, Ibn Rochd University Hospital, Casablanca, MAR

**Keywords:** graves´disease, hyperthyroidism, autobiographical case report, dexamethasone, neutropenia

## Abstract

Severe neutropenia in newly diagnosed hyperthyroidism is a diagnostic and therapeutic dilemma since antithyroid drugs (ATDs) cannot be started if the absolute neutrophil count (ANC) is <1 x 10^9^/L. We report the case of a patient followed for hyperthyroidism associated with severe neutropenia treated with dexamethasone and ATD. The patient was 51 years old and was hospitalized for hyperthyroidism with a thyroid stimulating hormone (TSH) level <0.005 (0.4-4) mUI/L, T4 at 415 (9.3-17.1) ng/L and T3 at 148 (2-4.4) pg/mL on Graves' disease (GD) confirmed by the TSH receptor antibodies at 38 IU/mL and scintigraphy, associated with neutropenia, with ANC at 0.4 x 10^9^/L. He was put on prednisolone 60 mg/day and propranolol 60 mg/day for three weeks without improvement. Faced with the association of hyperthyroidism and severe neutropenia, we could not start the ATD for fear of agranulocytosis; we put the patient on propranolol 60 mg and dexamethasone 6 mg with progressive degression resulting in a spectacular increase of ANC from 0.4 x 10^9^/L to 7.1 x 10^9^/L, which allowed us to start the ATD (carbimazole) at a dose of 30 mg, and then 50 mg, with monitoring of ANC and transaminases every 48 hours. Euthyroidism was achieved after 15 days. A curative treatment with radioactive iodine ablation was administered. Our patient did not respond to prednisolone but responded dramatically to dexamethasone; this leads us to consider using dexamethasone for the rapid preparation for radical treatment of patients with GD.

## Introduction

Graves' disease (GD) was first described by the Irish physician Robert James Graves in 1835 [1]. It is a specific autoimmune disease of the thyroid gland affecting approximately 12% of the population, with a female preponderance. It is characterised by the presence of TSH receptor antibodies (TRAb) and T and B lymphocyte infiltration [2]. The mechanism that triggers the autoimmune response remains unclear, but a genetic predisposition interacting with environmental factors may be involved [3].

Treatment modalities for GD include prescription of antithyroid drugs (ATDs), surgery and radioactive iodine (RAI) therapy with iodine-131 (131I). The choice of treatment depends on several factors such as severity of thyrotoxicosis, size of goiter, response to treatment and presence of other comorbidities [4]. However, the treatment of first choice remains the ATDs, which have the effect of restoring euthyroidism [5-6].

ATDs are an effective and safe therapy for GD hyperthyroidism, but contraindications and adverse reactions may limit their use, including severe neutropenia, which is a well-known adverse effect of ATDs, and which may occur during treatment of hyperthyroidism [7]. This neutropenia may also be a sign of newly diagnosed hyperthyroidism, which is why the American Thyroid Association suggests that a complete blood count should be checked before starting treatment with ATDs and that they should not be started when the absolute neutrophil count (ANC) is <1 x 10^9^/L [4]. Therefore, the discovery of severe neutropenia in newly diagnosed hyperthyroidism is a major diagnostic and therapeutic dilemma.

We report the case of a patient admitted for severe hyperthyroidism associated with severe neutropenia treated successfully with dexamethasone and the ATD.

## Case presentation

The patient was 51 years old, admitted for hyperthyroidism, with a history dating back to nine months before admission, onset of a weight loss of 20 kg contrasting with polyphagia associated with palpitation, tremors of extremities and digestive disorders such as motor diarrhea. Clinical examination revealed a blood pressure of 150/70 mmHg, tachycardia at 100 bpm, temperature of 37°C, weight 65 kg with a weight loss of 20 kg, and bilateral exophthalmos with European Group on Graves’ Orbitopathy (EUGOGO) stage 1 orbitopathy without pretibial myxedema. Cervical examination revealed a homogeneous, soft, pulsatile, grade 1 goiter with no irregularities and free lymph nodes.

Biological assessment on admission showed hyperthyroidism with a thyroid stimulating hormone (TSH) level <0.005 (normal 0.4-4) mU/L, T4 elevation at 415 (normal 9.3-17.1) ng/L and T3 at 148 (normal 2-4.4) ng/L. The blood count showed neutropenia with ANC at 0.4 x 10^9^/L (normal 2-7 x 10^9^/L).

In the etiological work-up of hyperthyroidism, TRAb were elevated at 38 UI/mL and scintigraphy showed a goiter under endogenous stimulation in favour of GD.

The patient was put on prednisolone 60 mg and propranolol 60 mg/day for three weeks before hospitalisation without improvement. We admitted him for the management of hyperthyroidism in the context of GD associated with severe neutropenia for which a myelogram was performed showing a rich marrow with numerous megakaryocytes. All lineages were represented with marked dysgranulopoiesis. A bone marrow biopsy also showed a normal rich marrow with non-specific maturation disorders.

Given the severity of the hyperthyroidism and the very high T4 levels, a paraneoplastic syndrome had to be ruled out, which is why the tumor marker assay (human chorionic gonadotropin, lactate dehydrogenase) was performed and was negative.

Given the combination of severe hyperthyroidism and severe neutropenia, we were unable to start ATDs for fear of agranulocytosis and scheduled plasmapheresis sessions in preparation for curative treatment.

**Figure 1:**
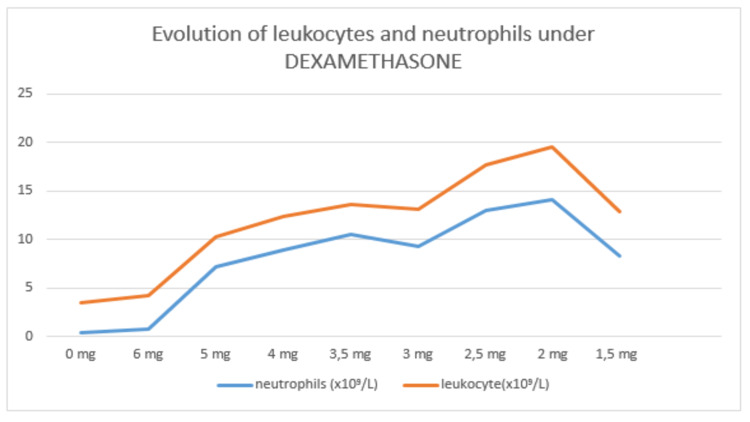
Evolution of leukocytes and neutrophils under dexamethasone

**Figure 2:**
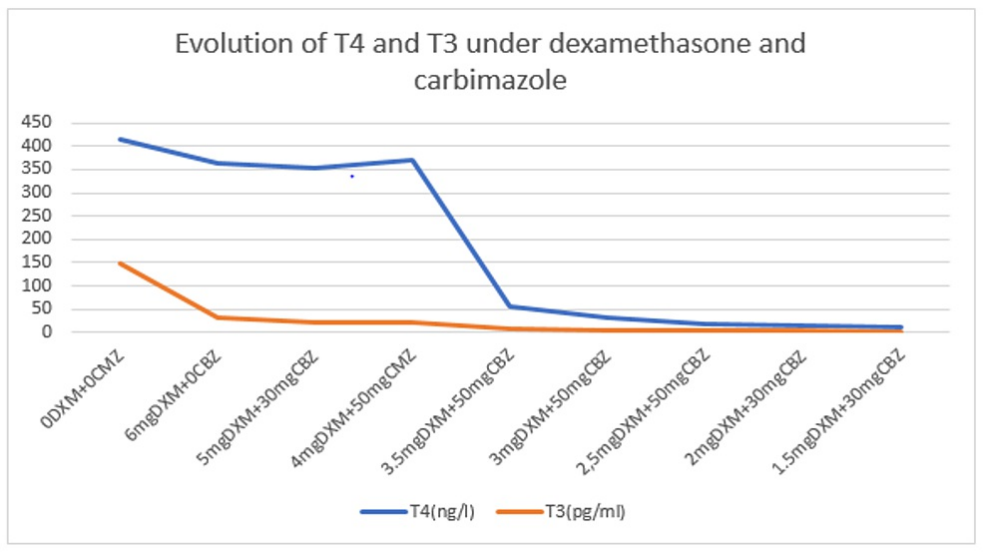
Evolution of T4 and T3 under dexamethasone and carbimazole DXM: dexamethasone; CBZ: carbimazole

While waiting for the response of the health care system with regard to plasmapheresis, we put the patient on propranolol 60 mg and dexamethasone 6 mg resulting in an increase of ANC from 0.4 x 10^9^/L to 7.1 x 10^9^/L, which allowed us to start the ATD (carbimazole) at a dose of 30 mg with monitoring of ANC and transaminases every 48 hours (Table 1). We increased carbimazole to 50 mg while decreasing dexamethasone until it was stopped (Figure 1). Thyroid hormones decreased progressively until normalization at day 10 of carbimazole (Figure 2).

**Table 1:** Evolution of biological parameters under dexamethasone and carbimazole DXM: dexamethasone; CMZ: carbimazole; ANC: absolute neutrophil count; T3: triiodothyronine; T4: thyroxine; ALAT: alanine aminotransferase; ASAT: aspartate aminotransferase

Date (February 2021)	DXM (mg)		CMZ (mg)	Leukocytes (4-10 x 10^9^/L)	ANC (2-7 x 10^9^/L)	T3 (2-4.4 pg/mL)	T4 (9-17 ng/L)	ALAT (<50 UI/L)	ASAT (<50 UI/L)
01		0	0	3.490	0.40	148	415	39	46
03		6	0	4.200	0.76	30.8	364	35	43
06		5	30	10.270	7.130	23	354	25	43
08		4	50	12.400	8.930	20.8	370	21	60
10		3.5	50	13.580	10.500	6.7	57	17	55
12		3	50	13.080	9.300	3.9	32	16	51
14		2.5	50	17.730	12.930	3.2	19	18	43
16		2	30	19.590	14.050	2.8	14	30	73
18		1.5	30	12.840	8.260	2.1	10	25	69
22		1	20						

Curative treatment with 10 MCi (370 MBq) of radioactive iodine ablation was administered after euthyroidism under corticosteroid coverage to avoid aggravation of the ophthalmopathy. 

Evolution

After curative treatment with radioactive iodine ablation, thyroid balance was checked once a week for the first month and then once a month. After two months, the patient gained 18 kg and presented hypothyroidism for which a substitution with levothyroxine was started.

## Discussion

The association between neutropenia and hyperthyroidism was first described by Caro in 1907 and confirmed one year later by Koche who proposed the triad of leukopenia, neutropenia and lymphocytosis as an early diagnosis of Graves' disease [8,9]. Subsequently, various studies were conducted to investigate the relationship between hyperthyroidism and neutropenia.

Coexistence of severe neutropenia (ANC ≤0.5 x 10^9^/L) with GD poses diagnostic and therapeutic challenges. It has been shown that neutropenia associated with GD is most often mild [10]. Therefore, other etiologies of severe neutropenia should be ruled out before simply linking it to GD. Several studies have found an inverse correlation between thyroid hormone level and neutrophil count, so more severe hyperthyroidism appears to be a predictor of neutropenia [11,12].

In cases of GD with severe neutropenia, curative treatment with radioactive iodine or surgery can be considered but due to the high thyroid hormone levels, the risk of acute thyrotoxic crisis is not negligible.

Our patient had severe neutropenia prior to the initiation of treatment; the prescription of an ATD was not possible for fear of inducing agranulocytosis. And the introduction of a curative treatment was dangerous because the risk of acute thyrotoxic crisis was not negligible in view of the very high level of free T4.

Moreover, our patient did not respond to prednisolone but responded spectacularly to dexamethasone, which led us to think of using dexamethasone in Graves’ ophthalmopathy and rapid preparation for the radical treatment of patients with GD, even if there exist no studies on this subject as per our knowledge.

Mechanism of neutropenia in hyperthyroidism in GD

The mechanism of neutropenia in GD remains unclear, but different theories could explain the close relationship between GD hyperthyroidism and neutropenia. Excessive thyroid hormones seem to significantly affect the proliferative potential of haematopoietic progenitor cells [13].

Some studies have suggested an autoimmune basis and shortened survival of peripheral neutrophils. The study by Weitzman et al. provided evidence that TRAb, and "classical" antineutrophil antibodies, may bind to the TSH receptor on neutrophils so that they can mediate neutrophils in some patients with GD [14]. The reduction in circulating neutrophils could be due to abnormal distribution and marginalisation, as shown in an animal study [15,16]. Adhesion molecules such as E-selectin promote the adhesion and aggregation of leukocytes to blood vessel walls. In one study, E-selectin levels were higher in patients with leukopenia than in GD patients with normal leukocytes. In addition, there was a negative correlation between leukocyte count and E-selectin levels. Interestingly, glucocorticoid (prednisone) treatment of patients with leukopenia resulted in a significant reduction in E-selectin levels [17].

Evolution of neutropenia

In a meta-analysis investigating the association between neutropenia and hyperthyroidism, in all patients with GD, treatment with ATDs and/or irradiation was also used to treat coexisting neutropenia [17]. The time to resolution of neutropenia in two studies was 14-55 days and occurred in parallel with the restoration of euthyroidism [10,18]. A similar time frame (60 days) was required to achieve the normalisation of severe neutropenia (ANC 0.2 × 10^9^/L) after treatment with carbimazole in a case study by Hegazi et al. [19].

Our patient did not respond to prednisolone but leukocyte levels increased dramatically after two days of dexamethasone that may have a more powerful effect on the demargination of neutrophils. The ANC continued to increase after the introduction of ATD and progressive normalisation of thyroid function.

## Conclusions

The combination of hyperthyroidism in GD and severe neutropenia is a diagnostic and therapeutic challenge. The introduction of an ATD for an ANC < 1 x 10^9^/L is not recommended by current guidelines. In our patient, dexamethasone produced a spectacular increase in the ANC after 48 hours that allowed for the introduction of the ATD and euthyroidism within 15 days. This suggests that a dose of dexamethasone can be given as a test treatment in patients with hyperthyroidism associated with GD and severe neutropenia after the diagnosis of malignancy has been ruled out.
